# Supplementary material for: Educator perceptions of the complex needs of young people in Pupil Referral Units: An exploratory qualitative analysis
Source: PLoS One. 2024 Sep 19;19(9):e0310633. doi: 10.1371/journal.pone.0310633 (PMC11412493; doi:10.1371/journal.pone.0310633)
Supplement: S1 File — (DOCX) [file pone.0310633.s001.docx]

**Demographics/ General**

| **Gender:** (Male/ Female/ Prefer not to say) | | **Position in Organisation/ Service/ Unit:** | |
| --- | --- | --- | --- |
| **Age:** | **Years in current role:** | | **Overall years worked in PRU setting:** |

| **Personal Experiences:**  *Q1. Can you describe your average day in the PRU?*   - If worked previously in a mainstream educational setting, how does it differ to your current experience   *Q2. How would you describe your working relationship with other agencies that are involved in the young person’s (YP) life?*   - Challenges/ Areas of improvement   *Q3. What do you see as the remit of your role in meeting the needs of the YP?* |
| --- |

| **Experiences with young people:**  *Q4. How would you describe the group of YP you are working with?*   - particular features of the cohort/ group - Appropriateness of being in the PRU (mix of YP within setting)   *Q5. What difficulties do you think YP encounter when coming to a PRU?*   - E.g. prior experience before coming to PRU, ACE, disruptive home, offending, care experience, previous experience in education - Observe any challenges in the transition from mainstream to PRU setting   *Q6. What is the psychological/ mental health background of the young people you are working with?*   - Have they been given any mental health diagnoses before they arrive at your placement? - If yes, what - Are there young people in your opinion that have a mental health condition that is not diagnosed? - What type of presentation   *Q6 Do the young people have regular input from a mental health professional?*   - If yes, who (what professional) / what service provided the input - What kind of input was it and how often   *Q7. Do you remember a YP’s case/ situation that required professional mental health input?*  *Q8. What do you understand to be a successful outcome for young people in a PRU?*   - Educational attainment/ Resilience building or should overall focus be on something different - situation where you believe you made a difference in a YP’s life/ situation |
| --- |

| **Experiences of the PRU practise provisions – Suggestions/ Opinion**  *Q9. What do you believe the young people require that is currently not provided?*  *Q10. What do you believe YOP themselves would identify what their needs are?*  *Q11. What support do you think you need to meet the needs of the YP?*  *Q12. Is there anything you would like to add?* |
| --- |
